# Supplementary material for: Phylogenetic Analysis and Emerging Drug Resistance against Different Nucleoside Analogues in Hepatitis B Virus Positive Patients
Source: Microorganisms. 2023 Oct 24;11(11):2622. doi: 10.3390/microorganisms11112622 (PMC10673510; doi:10.3390/microorganisms11112622)
Supplement: Supplementary file 1 [file microorganisms-11-02622-s001.zip › microorganisms-2642773-supplementary.pdf]

Table S1: Viral Load of all the study cohort.

Patient ID	Viral load IU/mL
1	5.2×10^4
2	3.2×10^6
3	3.5×10^6
4	3.5×10^4
5	3.0×10^4
6	5.6×10^4
7	3.6×10^6
8	3.8×10^4
9	5.4×10^5
10	4.6×10^4
11	6.4×10^6
12	5.0×10^4
13	5.2×10^4
14	3.3×10^4
15	4.8×10^4
16	3.5×10^5
17	4.2×10^4
18	3.6×10^5
19	6.0×10^4
20	5.1×10^6
21	3.2×10^5
22	5.6×10^5
23	6.8×10^5
24	4.5×10^5
25	3.4×10^6
26	5.6×10^7
27	5.3×10^3
28	4.0×10^3
29	3.2×10^6
30	3.0×10^4
31	3.9×10^6
32	3.0×10^5
33	6.3×10^4
34	5.2×10^6
35	4.4×10^4
36	4.9×10^4
37	3.3×10^5
38	3.8×10^4
39	4.8×10^5
40	4.0×10^4

Table S2: Sequences generated from current study along with accession numbers and urls.

S.no	Submission ID	Accession Number	URLS
1	BankIt2162064 Seq1	MK213855	https://www.ncbi.nlm.nih.gov/nuccore/MK213855
2	BankIt2162064 Seq2	MK213856	https://www.ncbi.nlm.nih.gov/nuccore/MK213856
3	BankIt2162064 Seq3	MK213857	https://www.ncbi.nlm.nih.gov/nuccore/MK213857
4	BankIt2162064 Seq4	MK213858	https://www.ncbi.nlm.nih.gov/nuccore/MK213858
5	BankIt2162064 Seq5	MK213859	https://www.ncbi.nlm.nih.gov/nuccore/MK213859
6	BankIt2162064 Seq6	MK213860	https://www.ncbi.nlm.nih.gov/nuccore/MK213860
7	BankIt2162064 Seq7	MK213861	https://www.ncbi.nlm.nih.gov/nuccore/MK213861
8	BankIt2162064 Seq8	MK213862	https://www.ncbi.nlm.nih.gov/nuccore/MK213862
9	BankIt2162064 Seq9	MK213863	https://www.ncbi.nlm.nih.gov/nuccore/MK213863
10	BankIt2162064 Seq10	MK213864	https://www.ncbi.nlm.nih.gov/nuccore/MK213864
11	BankIt2162064 Seq11	MK213865	https://www.ncbi.nlm.nih.gov/nuccore/MK213865
12	BankIt2162064 Seq12	MK213866	https://www.ncbi.nlm.nih.gov/nuccore/MK213866
13	BankIt2162064 Seq13	MK213867	https://www.ncbi.nlm.nih.gov/nuccore/MK213867
14	BankIt2162064 Seq14	MK213868	https://www.ncbi.nlm.nih.gov/nuccore/MK213868
15	BankIt2162064 Seq15	MK213879	https://www.ncbi.nlm.nih.gov/nuccore/MK213869
16	BankIt2162064 Seq16	MK213870	https://www.ncbi.nlm.nih.gov/nuccore/MK213870
17	BankIt2162064 Seq17	MK213871	https://www.ncbi.nlm.nih.gov/nuccore/MK213871
18	BankIt2162064 Seq18	MK213872	https://www.ncbi.nlm.nih.gov/nuccore/MK213872
19	BankIt2162064 Seq19	MK213873	https://www.ncbi.nlm.nih.gov/nuccore/MK213873
20	BankIt2162064 Seq20	MK213874	https://www.ncbi.nlm.nih.gov/nuccore/MK213874
21	BankIt2162064 Seq21	MK213875	https://www.ncbi.nlm.nih.gov/nuccore/MK213875
22	BankIt2162064 Seq22	MK213876	https://www.ncbi.nlm.nih.gov/nuccore/MK213876
23	BankIt2162064 Seq23	MK213877	https://www.ncbi.nlm.nih.gov/nuccore/MK213877
24	BankIt2162064 Seq24	MK213878	https://www.ncbi.nlm.nih.gov/nuccore/MK213878
25	BankIt2162064 Seq25	MK213879	https://www.ncbi.nlm.nih.gov/nuccore/MK213879
26	BankIt2162064 Seq26	MK213880	https://www.ncbi.nlm.nih.gov/nuccore/MK213880
27	BankIt2162064 Seq27	MK213881	https://www.ncbi.nlm.nih.gov/nuccore/MK213881

28	BankIt2162064 Seq28	MK213882	https://www.ncbi.nlm.nih.gov/nuccore/MK213882
29	BankIt2162064 Seq29	MK213883	https://www.ncbi.nlm.nih.gov/nuccore/MK213883
30	BankIt2162064 Seq30	MK213884	https://www.ncbi.nlm.nih.gov/nuccore/MK213884
31	BankIt2162064 Seq31	MK213885	https://www.ncbi.nlm.nih.gov/nuccore/MK213885
32	BankIt2162064 Seq32	MK213886	https://www.ncbi.nlm.nih.gov/nuccore/MK213886
33	BankIt2162064 Seq33	MK213887	https://www.ncbi.nlm.nih.gov/nuccore/MK213887
34	BankIt2162064 Seq34	MK213888	https://www.ncbi.nlm.nih.gov/nuccore/MK213888
35	BankIt2162064 Seq35	MK213889	https://www.ncbi.nlm.nih.gov/nuccore/MK213889
36	BankIt2162064 Seq36	MK213890	https://www.ncbi.nlm.nih.gov/nuccore/MK213890
37	BankIt2162064 Seq37	MK213891	https://www.ncbi.nlm.nih.gov/nuccore/MK213891
38	BankIt2162064 Seq38	MK213892	https://www.ncbi.nlm.nih.gov/nuccore/MK213892
39	BankIt2162064 Seq39	MK213893	https://www.ncbi.nlm.nih.gov/nuccore/MK213893
40	BankIt2162064 Seq40	MK213894	https://www.ncbi.nlm.nih.gov/nuccore/MK213894

Table S3: Samples with no drug resistance mutations.

S. No	Drug resistance mutations	Therapy Used	Small Surface Gene Mutation
1	Not detected	Tenofovir	Not detected
2	Not detected	Adefovir	Not detected
3	Not detected	Tenofovir	Not detected
4	Not detected	Tenofovir	Not detected
5	Not detected	Tenofovir	Not detected
6	Not detected	Entecavir	Not detected
7	Not detected	Adefovir	Not detected
8	Not detected	Tenofovir	Not detected
9	Not detected	Lamivudine+ tenofovir	Not detected
10	Not detected	Lamivudine+ tenofovir	Not detected
11	Not detected	Tenofovir	Not detected
12	Not detected	Adefovir	Not detected
13	Not detected	Lamivudine+ tenofovir	Not detected
